# Empyema of the Antrum

**Published:** 1898-08

**Authors:** Emma E. Musson


### EMPYEMA OF THE ANTRUM.[1]

BY DR. EMMA E. MUSSON.[2]

Mr. President and Members of the Society,—The anatomical relation of the roots of the teeth to the floor of the antrum will determine, to a great extent, the involvement of the sinus in various dental affections. Between the floor of the sinus and the roots of the teeth there may intervene a thick layer of bone, or this layer may be reduced to a mere line, or the roots of the teeth may protrude directly into the antrum.

In a case recently reported, of necrosis of the floor of the antrum following upon destruction of nerve-roots with arsenic, this dividing line must have been very slight. With the appearance of la grippe, the number of cases of inflammatory conditions of the accessory sinuses have been greatly on the increase, and a tendency has arisen to attribute maxillary sinusitis to nasal causes. Mr. Wolff, of Germany, in a series of post-mortem examinations, found in the majority of the cases of measles, scarlet fever, and diphtheria, some degree of inflammation of the accessory sinuses,—a point of great interest in connection with Moure's and Ziem's statements, that they have frequently found empyema of the antrum in children of seven years and over.

Very little work has been done, as yet, in the line of bacteriological examination of the character of the pus in diseases of the accessory sinuses, or of microscopical work as to pathological conditions of the mucous membrane of the antrum. Clearer knowledge on these points, as on that of the anatomical conformation of

---

[1] Read before the Odontological Society of Pennsylvania, February 12, 1898.

[2] Clinical Professor of Laryngology of the Woman's Medical College, Philadelphia.

the antrum and its relations to the ethmoidal and frontal sinuses, would materially lessen the present apparent divergence of opinion as to prognosis and the results of treatment.

Of twenty cases of abscess of the antrum collected from my books, eleven were chronic cases and nine acute; of the chronic conditions, in only two were the teeth removed as a possible cause of the empyema, and in one of these there was a clear history of infection following packing for nasal hemorrhage. In five no history could be gained; however, four of these cases dated back ten years and over. Three cases commenced with la grippe, and the eleventh case occurred in a patient suffering from pulmonary phthisis.

In the nine patients in which the affection was acute, one accompanied a severe cold in the head; one followed an acute infectious naso-pharyngitis; one, a nasal cauterization during the prevalence of la grippe; five were due to la grippe; and in the ninth case, in which the ethmoidal and frontal sinuses were also involved no possible cause could be assigned for it. The patient, a university student, had had, three weeks previously, a severe neuralgic pain in the left side of the face. The pain was attributed to a lower molar, and the nerve was destroyed, but without relief. After three weeks fever set in, and there was a free discharge of pus from the left nostril. At this time the patient came under my care and the above diagnosis was made. Though a month had elapsed before treatment was instituted, the inflammation of the accessory sinuses yielded rapidly to treatment, the main feature of which was the inhalation of oxygen through the left nostril.

In five of the chronic and in four of the acute cases of empyema of the antrum there was a coexisting inflammatory condition of the ethmoidal cells, or of the frontal sinus, or of both.

An acute abscess of the antrum may be usually diagnosed by its symptoms. The sudden onset of neuralgic pain in the face, which abates somewhat on the appearance of a profuse discharge of blood and pus from the nostril of the side affected, tenderness on pressure over the canine fossa, and fever are sufficient to warrant a diagnosis. As to pain as a symptom, Hajek thinks it is greatest with an antrum with a thin floor, as the dental nerves are thus in close relation to the mucous membrane of the sinus. A unilateral discharge of pus from the nose, flowing more freely when the head is depressed, is sometimes the only symptom that a patient with a chronic antral sinusitis complains of. If on anterior rhinoscopic examination pus is found oozing from beneath the middle turbinate and on the same side the face remains dark on using the transillumination test of Voltolini, our diagnosis is sufficiently established to warrant surgical interference. I think the failure of the electric lamp to establish a diagnosis is due, except where there are anatomical abnormalities, to a lamp of too small candle-power or to a battery of insufficient voltage. I do not consider this test a final one unless the pupil of the unaffected side is thoroughly illuminated, although the majority of the text-books teach that translucency of the tissues in the infraorbital region excludes empyema of the antrum.

To illustrate this point: a patient with fair hair and skin and small-boned was suffering from a unilateral flow of pus from the left nostril. On examination with electric light there was no shadow in the infraorbital regions; the whole face was perfectly translucent, but the light was seen shining through the right pupil only; the left remained perfectly dark. After drilling through the left alveolus, the teeth being absent, a half ounce of foul-smelling pus was syringed out. A month later both pupils could be illuminated.

By means of the electric lamp a diagnosis can be made from a cyst of the antrum, as the side affected is more brilliantly illuminated where there exists cystic disease.

A cure of abscess of the maxillary sinus is not always followed by a complete disappearance of the shadow in the infraorbital region on transillumination, as a thickened mucous membrane may still remain. The first point at which the translucency of the tissues begins to reappear is at the inner angle of the orbit, gradually extending outward till the whole infraorbital region is clear.

Anatomical abnormalities will complicate the correctness of Voltolini's test. The orbital plate may be thick and dense; the antrum itself may be small or rudimentary, or its walls compressed laterally, or the external wall of the inferior meatus may encroach on its lumen. In the negro race the pigment interferes with the transmission of the light.

Lichtwitz, as a diagnostic measure, punctures the inferior meatus, using a very fine trocar and canula of his own design. Gavel diagnoses antral abscess by syringing it out through the ostium maxillare, having succeeded in passing the catheter in twenty-eight cases out of forty. The canula used is according to Heryng's pattern, with bent tip. It is introduced with curve downward in the direction of the middle meatus. Having passed the anterior portion of the middle turbinate, the curved extremity of the canula is passed outward and the hiatus semilunaris is gently explored; the sound becomes suddenly engaged in a small cavity which does not permit of it being moved either backward or forward. Zuckerkandl states that the ostium maxillare is profoundly hid by the inferior lip of the hiatus semilunaris, and even if a probe is introduced into the opening it is exceedingly difficult to push it anteriorly into the cavity of the sinus, as the ostium forms a canal whose direction is from above downward and from behind forward, —exactly opposite to that of an instrument penetrating into the middle meatus. In the specimens I have examined and show here this evening, preliminary removal of the anterior portion of the middle turbinate would be necessary to make possible the syringing out of the antrum through the ostium.

In acute infections of the antrum, whatever the cause, much can be done to shorten the attack and lessen the danger of its becoming chronic. The primary necessity is to establish good drainage, first, by keeping the ostium maxillare patulous by such means as will allay inflammation of the mucous membranes of the nasal cavities and the antrum, and in the second place by employing various methods for emptying the antrum of pus, directing the patient to lie on the non-affected side with the head dependent over the edge of the bed, and several times during the day practise aspiration of the contents of the antrum according to the method of Dr. Moll, given at the last annual meeting of the Dutch Laryngological Society. It consists of the aspiration of the contents of the accessory cavities by a forcible inspiration, while the mouth and nose are firmly closed. In this way the chest is inflated and there is a considerable diminution of pressure in the accessory cavities, and the liquid contents are drawn out, as is proved by the reappearance of pus in the middle meatus after cleansing. In a case in which there was a perforation of the alveolus Dr. Moll proved a diminution of pressure of fifteen millimetres.

The use of oxygen in inflammatory conditions of the accessory sinuses was first suggested to my mind by Mr. Stokes's (of London) enthusiastic advocacy of it in chronic abscess of the middle ear. Later I found he was applying it directly to the antrum in abscess of that sinus, and intends publishing his results. The first case in which I used it was in one of acute infection of the accessory sinuses. The abscess of the antrum resisted all treatment, and, in consultation, opening up of the antrum was advised. As a last resort I prescribed oxygen to be inhaled for fifteen minutes every four hours, and through the affected side. In a few days the discharge was muco-purulent only, and then mucoid. In this case, after recovery, the affected side of the face failed to clear up on electric illumination.

In chronic cases, where there is a diseased tooth, or where one of the molars or the second bicuspid is gone, I invariably drill up through the alveolar process, making an opening of about four millimetres in diameter. The advantages of the alveolar or Cooper's operation over that through the canine fossa or inferior meatus—the latter Mikulicz's method—is that the antrum is opened at its lowest point, and can be treated more thoroughly and with least pain, both by doctor and patient. In favor of this method would be Zuckerkandl's statement that he found that in most of his specimens that the sinus dipped very low into the alveolar border in empyema of the antrum on account of the most intimate relation with the dental roots. Ziem, of Dantzig, sometimes drills between the teeth on the palatal surface, using very fine burs. He acknowledges that one time he missed the sinus.

There are several anatomical anomalies where Cooper's operation would not be advisable,—for example, where there is a thick alveolar process. Zuckerkandl states that a normal maxillary sinus terminates inferiorly on a horizontal plane passing through the floor of the nasal fossa; therefore, the higher the palatine vault the higher the floor of the sinus will be situated and, in consequence, the thicker will be the alveolar border.

The alveolar operation would not be advisable where there is a flattening of the antrum laterally. Hajck has observed that in these cases there is usually an exaggeration of the concavity of the external wall of the inferior meatus corresponding to the effacement of the canine fossa. In one case, where the alveolar ridge was very thin from long absorption, I commenced the drilling above the edge and on the buccal surface of the alveolus.

When the teeth are perfectly sound I select the canine fossa for the point of operation, making an opening about three millimetres in diameter, directing the bur upward, backward, and slightly inward, having previously incised the mucous membrane. The opening is very rapidly made, as the wall of the antrum is here very thin. The bleeding and the pain are slight in comparison with that in the operation through the inferior meatus. It is also advisable where there is reason to suspect that a more extensive operation will have to be performed for curettement and removal of polypoid growths. Its disadvantages are the temporary swelling of the face after operation, and that syringing here is more painful than through an alveolar opening.

Zuckerkandl, as the result of his anatomical studies, advises opening into the maxillary sinus through the inferior meatus, puncturing as far posteriorly as possible where the wall is thin and immediately below the line of insertion of the inferior turbinate. Dr. Freeman, of Philadelphia, has met with much success with this method of treatment. The difficulty with which the after-treatment is carried out seems to me the greatest objection to this operation. The operation itself is attended with considerable hemorrhage, and where the nostril is narrowed from deviation or spur of the septum, opening up the antrum, either by trocar or drill, becomes difficult if not impossible. The naso-antral wall may be so thick that a bur would penetrate with difficulty, and Krause's trocar not at all.

Sometimes the antrum is situated high up and backward, when an opening made in the inferior meatus would in all probability penetrate into the canine fossa, or if the antrum is narrow, even if the puncture is made far back, the drill may pass through the antrum and into the canine fossa. This narrowness, as before stated, may be suspected where there is an exaggerated concavity of the wall of the inferior meatus, or, according to Gougenheim, where there is marked flattening of the palatine vault.

After opening up the antrum the cure of the sinusitis will depend greatly upon the thorough and regular flushing out of the cavity. I find it is advisable to have a detachable canula that remains in the sinus during the syringing. After this has been done I get rid of the solution left in the cavity by injecting air, either by means of the syringe or a Politzer bag, and then insufflate powder. If the case promises to be of short duration, an ivory plug is introduced; otherwise, a tube of silver or platinum.

If empyema does not yield to these measures, then it will be advisable to make a large opening through the canine fossa to treat a possible necrosis or polypoid growth. Luc completes this operation with a counter-opening made through the inferior meatus with a Krause's trocar, introduces a tube, and allows the canine fossa to heal over. He claims for this method rapidity of cure.

After the sinus is curetted it should be tightly tamponed with iodoform gauze, which should be allowed to remain in for forty-eight hours.

In the first two cases in which I curetted the antrum to remove polypoid growths I chiselled away the anterior wall of the antrum; in the third case Dr. Cryer kindly operated for me, with burs and handle of his own invention. The operation was done with great rapidity, and with much less traumatism and hemorrhage.

Scheich makes a large opening through the alveolus, and packs with gauze two centimetres in width and with a selvage on both edges.

At birth the maxillary sinus is merely a small opening, extending laterally. From this time until seven and a half years of age, according to Bourgeois, these cavities become notably enlarged. During this period the relation of the teeth to the antrum is variable; for example, the relation of the second premolar to the antrum of Highmore in an infant at term is much nearer than in a child at seven and a half. Moure, in an article on "Empyema of the Antrum in Children," quotes two unique cases of antral abscess in infants three weeks old,—in both there was a premature eruption of a tooth,—and states that this affection is quite common in children of seven years of age and over.

In a specimen I will show to-night we have both the sinuses encroached upon by an excessive concavity of the walls of the inferior and the middle meatus. This lack of absorption sometimes shows itself in the antrum by the presence of bony or membranous partitions.

The anomalous openings that have been found to exist between the frontal and antral sinuses have given rise to the question whether some of our intractable cases of empyema of the antrum are not due to a frontal abscess discharging itself through the antrum. Dr. Fillebrown, of Boston, gives a diagrammatic drawing of the infundibulum, continuing as a half tube, which terminates directly in the foramen of the maxillary sinus. This half tube, I suspect, is what the French term the gouttière de l'infundibulum, or groove of the infundibulum, to distinguish it from the infundibulum proper. Paul Raugé writes that the term infundibulum, meaning a funnel, should be reserved for that canal that passes from the frontal sinus through the anterior ethmoidal cells; this canal at its narrowest portion becomes continuous with the apex of another infundibuliform canal,—the gouttière de l'infundibulum,—the whole having the form of an hour-glass. The canal of the infundibulum, however, is not, like the infundibulum proper, a complete cavity, as on its upper portion it is open its full length,—the hiatus (or cleft) semilunaris of Zuckerkandl. It is a channel formed in the external wall of the middle meatus, having for its upper boundary the bulla ethmoidalis and for its inferior edge the unciform process.

In the floor of this groove of the infundibulum, below and anterior to the bulla ethmoidalis, is to be found the constant opening into the maxillary sinus. The opening is so situated in the floor of a narrow channel that, as Raugé writes, it is much better disposed to lead into it liquids flowing from the frontal sinus than to evacuate into the meatus those which come from the antrum.

Clinically, I have very frequently found associated abscess of the antrum with ethmoidal disease. Curnow, of King's College, London, has occasionally found that a direct communication exists between both the anterior and posterior ethmoidal cells and the summit of the antrum. I am inclined to think that ethmoiditis plays a very important *rôle* as a primary source of infection both in empyema of the antrum and the frontal sinus,—in the case of the antrum by flow of pus into it through anomalous openings, and in that of the frontal sinus by extension of inflammation through fronto-ethmoidal cells and the blocking of the infundibulum by polypoid masses.

